# Nutritional and health status of adult Syrian refugees in the early years of asylum in Germany: a cross-sectional pilot study

**DOI:** 10.1186/s12889-022-14684-7

**Published:** 2022-11-29

**Authors:** Feras Al Masri, Mattea Müller, Dorothee Straka, Andreas Hahn, Jan Philipp Schuchardt

**Affiliations:** 1 Institute of Food Science and Human Nutrition, Gottfried Wilhelm Leibniz University of Hannover, Am Kleinen Felde 30, 30167 Hannover, Germany; 2 Osnabrück University of Applied Sciences, Agricultural Sciences and Landscape Architecture, Osnabrück, Germany

**Keywords:** Syrian, Nutritional status, Refugees, Dietary intake

## Abstract

**Background:**

Migration is usually accompanied by changes in the social, cultural, and religious environment, socioeconomic status, and housing conditions, all of which affect nutritional health. In a cross-sectional study, we assessed the dietary intake as well as nutritional and health situation in a population of Syrian refugees who have resided in Germany for at least six months up to four years since 2015. The primary aim of this pilot study was to evaluate the nutritional and health status in comparison to reference values.

**Methods:**

Between December 2018 and March 2020, 114 adult Syrian refugees were included in the study. The subjects filled out questionnaires on sociodemographic variables, exercise, and nutrition behavior (three-day nutrition record). After a fasting blood draw, the subjects were examined for anthropometric parameters (height, weight, body mass index, waist circumference, waist-hip ratio, and body composition via a bioelectrical impedance analyzer). Various blood markers including iron status, hematological parameters, Vitamin D status, lipid metabolism, glucose metabolism, and total homocysteine (tHcy) were measured.

**Results:**

About half of the participants (71 male, 43 female) had lived in Germany for less than three years. Over 60% of men and 30% of women were overweight (BMI 25–30 kg/m2) or obese (BMI > 30 kg/m2), while 79% of men and 74% of women observed an elevated body fat mass. The evaluation of the three-day nutrition records revealed an unfavorable supply situation for numerous critical nutrients. More than half of the women (53.5%) had depleted iron stores (serum ferritin < 15 µg/l). The 25-OH-Vitamin D blood levels showed a high prevalence of Vitamin D insufficiency (25–49.9 nmol/l: 38% of men and 21% of women) and deficiency (< 25 nmol/l: 44% of men and 70% of women). 83% of men and 67% of women showed tHcy levels in plasma > 10 nmol/l. Fasting insulin levels and the HOMA-IR index indicate a risk for insulin resistance. Hyperlipidemia was prevalent, especially in males with 24% showing hypertriglyceridemia (> 150 mg/dl) and LDL-hypercholesterolemia (> 130 mg/dl).

**Conclusions:**

The nutritional and health status of the cohort of Syrian refugees in Germany examined in this study is unsatisfactory, and many of the investigated refugees are at risk for developing cardiovascular disease and type 2 diabetes mellitus. Further studies are required to investigate the nutritional and health situation of refugees. This is obligatory to find ways to avoid malnutrition with all its associated health, sociodemographic, and economic consequences.

## Background

Germany is one of the European countries that reported more than 1.1 million refugees by January 2021. Among them are 700,000 Syrians who have been admitted since the beginning of the Syrian conflict in March 2011. This makes Syrian refugees the third largest group of foreigners in Germany [1–3]. The process of migration is associated with changes in the social, cultural, and religious environment. Likewise, there are shifts in socioeconomic status and housing conditions. The impact on migrants' nutritional health is undeniable [4–6]. From the beginning of the migration until the arrival in the host country, the refugees face nutritional challenges, which are often characterized by a lack of energy and protein intake as well as micronutrient deficiency [7–9]. Moreover, nutritional acculturation occurs during the residence in the host country alongside the length of stay [10]. In general, acculturation describes cultural, psychological, and nutritional changes that occur during migration from one culture to another [11]. Accordingly, dietary acculturation refers to the adaptation to the eating habits within the host country [7]. However, due to the dominance of energy-dense and highly processed foods in Western countries and the lack of nutritional education [5, 11], the prevalence of nutrition-related diseases such as obesity and type 2 diabetes mellitus (T2D) increases with the dietary acculturation of refugees in Western countries [12]. In North America, for example, obesity prevalence among Latino, African and Asian immigrants increased according to the length of their stay in the host country [13]. Furthermore, refugees of different ethnicities in the US reported increased consumption of meat and eggs after immigration. Meanwhile, the consumption of vegetables, fruits, and dairy products was related to socioeconomic factors, food insecurity, previous experience of food deprivation, length of stay, region of origin, and age [14–16].

As corresponding data prior to migration is not available, the question of whether the nutritional and health status of Syrian refugees has deteriorated or improved can currently not be answered. The nutritional situation in many areas of Syria itself is classified as poor [17]. Surprisingly, a study from 2016 with Syrian refugees in Iraq, Jordan, and Lebanon showed that global acute malnutrition was relatively low in the observed study populations [17]. However, the study also revealed that anemia was a common problem among women and children [17]. In addition, there is little data on the nutritional and health status of Syrian refugees in Europe. Previous studies conducted in Germany and Switzerland focused on risk factors related to overweight and obesity [7, 18] or on the development of obesity and associated diseases after resettlement [19]. Furthermore, the studies mainly focused on the health and nutritional needs of children rather than healthy adult refugees [20, 21].

In Germany, initial medical examinations of refugees are required by law, although with a focus primarily on infectious diseases. Unfortunately, the screening and treatment of malnutrition and undernutrition are marginal, thus little nutrition-related information is collected [22–24]. Nevertheless, a German study from 2019 indicated that the health status of Syrian refugees in Germany is generally good relative to refugees of other nationalities [25]. Another recent German study on the dietary behavior of Syrian refugees observed an increasing awareness of healthy eating and lifestyle during the first years of asylum in Germany. Although post-migration stress factors, lack of practical knowledge on how to prepare favoured dishes, and food insecurity in the new environment make it difficult for the refugees to achieve the preferred diet [28]. However, it has not been investigated whether this increased nutritional awareness also affects the nutritional and health status.

Therefore, this cross-sectional pilot study assessed dietary intake as well as nutritional and health status in a population of adult Syrian refugees who have resided in Germany for at least six months up to four years since 2015. The primary objective of the study was to evaluate nutritional and health status in comparison to reference values. The secondary objective was to examine associations between markers of nutritional and health status markers and duration of asylum.

## Methods

### Study design and study population

This cross-sectional study was conducted at the Institute of Food Science and Human Nutrition at Leibniz University Hanover between December 2018 and March 2020 and included 114 Syrian refugees. The study was ethically approved by the Ethics Committee of the University of Applied Sciences Osnabrück [26]. All participants gave their written informed consent. Data were collected and processed following the Lower Saxony Data Protection Act, adhering to the Declaration of Helsinki and the principles of Good Clinical Practice. The participants were recruited by announcing the study on social media, in the local press, in refugee reception centers, and in language schools that conduct the integration courses.

**Fig. 1:**
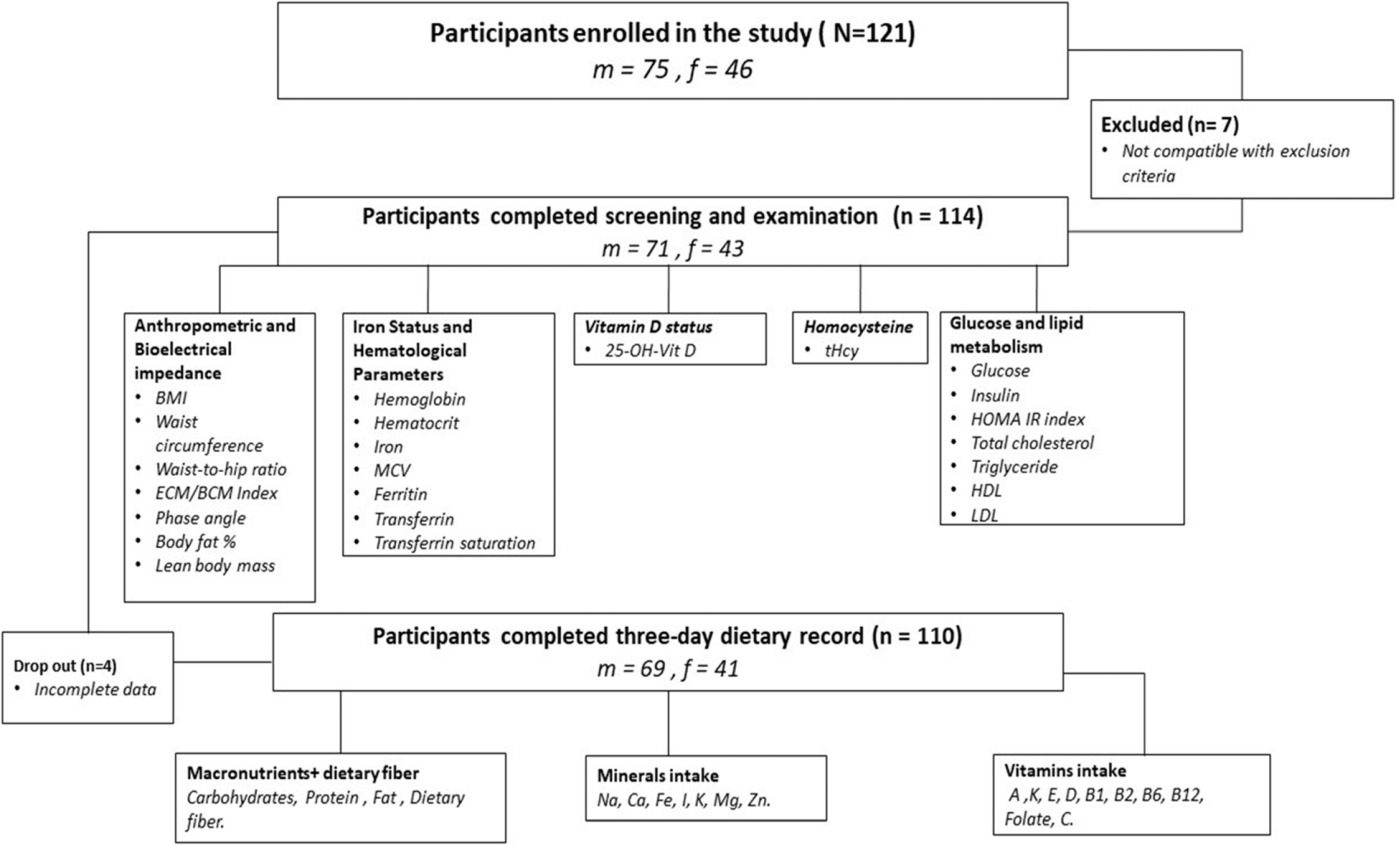
Flow chart of the study population

The suitability of the subjects to meet the inclusion criteria was determined by questionnaires. The participants were included if they had Syrian nationality and one of the following statuses: Asylum, Refugee Protection, or Subsidiary Protection, while other types of residence were not considered. Additional inclusion criteria were the age between 18 and 45 years, a residence in Hanover, Germany, and the surrounding areas, and an asylum status since 2015, the year in which a significant number of Syrian refugees arrived in Germany [27]. Cardiovascular, metabolic or malignant diseases, gastrointestinal diseases, pregnancy, food intolerances, and drug or alcohol addiction were criteria that resulted in exclusion. The study population was selected to be representative of the entire refugee population that immigrated between 2015 and 2020 in terms of gender and age distribution, as reported by the Federal Office for Migration and Asylum in Germany (BAMF) [28–32]. During the period of the study, approximately 15,000 foreigners with Syrian nationality lived in the greater Hanover area [33]. Eligible participants were invited for clinical investigation, which included screening information, anthropometric measurements, and a fasting blood sample. Before the clinical examination, participants were asked to complete a three-day dietary protocol and a questionnaire on their daily dietary behaviour. The nutritional and health status was assessed by anthropometrics and bioelectrical impedance measurements, blood biomarkers, and food intake (Fig. 1).

### Anthropometrics and bioelectrical impedance measurement

Participants were asked to appear in the morning fasted (≥ 10 h without food or caloric beverages) for their examination and blood draw. Height and weight were measured to calculate the body mass index (BMI), which is the ratio of weight and height to the square. Waist circumference (Wc) was measured between the lowest rib and the highest hip bone at the narrowest part of the midsection using a tape measure. The waist-to-hip ratio (WHR) was calculated by measuring the hip circumference above the widest part of the hip and then dividing the Wc by the hip circumference. Body composition was analyzed using a bioelectrical impedance analyzer (BIA) (Nutriguard M, Data Input Company, Darmstadt, Germany) and NutriPlus© 5.4.1 software (Data Input Company, Darmstadt, Germany). The BIA markers extracellular mass index/body cell mass (ECM/BCM Index), phase angle (PA), body fat (BF), and Lean Body Mass (LBM) were assessed. For the measurements, the participants were instructed to lay down on a stretcher and rest for a few minutes to ensure a balanced distribution of body fluids before the measurement. During the measurement, participants were instructed to lay still and relaxed, with the limbs slightly bent from the torso. A trained nutritionist performed all measurements.

### Blood biomarkers and biochemical analyses

Fasting blood samples were drawn by venipuncture of the antecubital vein using EDTA, serum, and NaF monovettes (Sarstedt AG & Co. KG, Nümbrecht, Germany) for the analysis of biochemical parameters. All samples were centrifuged, stored at 5 °C, and then transferred on dry ice to an accredited and certified laboratory (Laborärztliche Arbeitsgemeinschaft für Diagnostik und Rationalisierung e.V., Hannover, Germany).

Hemoglobin was analyzed using the cyanide-free SLS method. The mean corpuscular volume (MCV) was analyzed via fluorescence flow cytometry (Sysmex Europe GmbH, Norderstedt, Germany). The hematocrit was determined by multiplying the mean corpuscular volume by the erythrocyte count. Further analysis were performed: Iron, uric acid, triglycerides, low-density lipoprotein cholesterol (LDL), and high-density lipoprotein cholesterol (HDL) were analyzed using a photometric method. Glucose was using a enzymatic UV test (hexokinase method). Ferritin and 25-OH-Vitamin D (25-OH-Vit D) were carried out via a chemiluminescent paramagnetic particle immunoassay method. Transferrin with an immunoturbidimetric method (Beckman Coulter GmbH, Krefeld, Germany).

Plasma tHcy was analyzed using high-pressure liquid chromatography (Chromsystems Instruments & Chemicals GmbH, München, Germany). In addition, the electrochemiluminescence immunoassay method (ECLIA) using Cobas 801e (Roche Diagnostics GmbH, Mannheim, Germany) was used to determine insulin. Homeostasis model assessment-estimated insulin resistance (HOMA-IR index) was calculated as follows: fasting insulin ^[µu/ml]^ * fasting blood glucose ^[mg/dl])^ / 405.

### Dietary intake and eating habits

Before the study, the participants filled out an Arabic questionnaire with multiple-choice questions about their daily eating habits. These included questions about the number of meals eaten daily, where the meals were eaten, the type of diet (i.e., halal, non-halal), the reason for the choice of diet, and whether the diet was similar to the pre-immigration diet. In addition, a three-day dietary protocol was completed prior to the day of the clinical investigation, which included two consecutive weekdays and one weekend day. The nutrition software PRODI6.4® (Nutri-Science GmbH, Freiburg, Germany) was used to analyze the amount of food as well as nutrient-specific data such as the content of energy, macronutrients, minerals, and vitamins in the reported diet over three days. The traditional Syrian meals were recorded with their detailed ingredients in the form of a recipe and integrated into the data of the PRODI6.4 nutrition software.

### References values

The anthropometric measures BMI, Wc, and WHR were categorized according to the WHO [34]. The nutrient intake was compared to the reference values of the German, Austrian, and Swiss Nutrition Societies for healthy adults (D-A-CH reference values of nutrient intake) [35]. Cut-off points for iron status and hematological parameters as well as biomarkers of glucose and lipid metabolism were categorized according to the American Board of Internal Medicine (ABIM) [36]. Serum 25-OH-Vit D levels were assessed according to the Institute of Medicine reference values [37] and tHcy levels according to American Heart Association [38].

### Statistical analysis

The descriptive analysis of the study population included percentages of socio-demographic characteristics and dietary habits. The results are presented as mean ± standard deviation (SD) or 95% confidence interval (CI). The non-normally distributed data were transformed with the natural logarithm function before multiple linear regression. The one-sample t-test was performed to compare the mean values of dietary intake with the D-A-CH reference values for nutrient intake. In the case of nutrients with a reference range between two values, the minimum value was calculated as the reference cut-off point. Multiple linear regression models were designed to compare the asylum duration with markers of nutritional status (anthropometric values, bioelectrical impedance, and blood biomarkers). All models were adjusted for the covariates of age, sex, monthly income, and, housing situation, as well as for eating habits, dietary patterns, dietary motive, daily meals, a home-like diet, and, in some cases, also with BMI. Statistical analysis was performed within SPSS software (IBM SPSS Statistics 26.0.0.0; Chicago, IL, USA). The values were used for statistical analysis for all domains and the significance level was set at *p* < 0.05.

## Results

### Characteristics and eating habits

Of the total 114 participants who completed screening and examination, 62.3% were male, and 37.7% were female (Table 1). About half of the participants had lived in Germany for less than three years; approximately half of the sample also had a university degree, and 14% had no education. Furthermore, more than 85% had an income of less than 1000 € income per month, and only 13% had between 1000 and 2000 € available from various sources such as unemployment benefits or work. In addition, about 89% of the participants lived in rented apartments, and less than 3% lived in refugee camps. It is worth noting that these data are comparable to the general situation of Syrian refugees in Germany, reported by the Federal Office for Migration and Asylum (BAMF) [1].

**Table 1:** Characterization of the study population

Socio-demographic factors n (%)				
**Sex**	**Asylum duration**		**Religion**	**Housing**
Male: 71 (62%)	< 1 year: 27 (24%)		Islam 95 (83%)	Rented house: 22 (19%)
Female: 43 (38%)	1 year—< 3 year: 32 (28%)		Christian 7 (6%)	Rented apartment: 89 (78%)
Total: 114	3 – 4 years: 55 (48%)		No Data 12 (1%)	Refugee camp: 3 (3%)
**Age groups (years)**	**Educational degree**		**Marital status**	**Personal monthly net income**
18 – 24: 35 (31%)	No education: 16 (14%)		Single: 80 (71%)	< 1000 €: 97 (85%)
25 – 29: 40 (35%)	Basic education: 7 (6%)		Married: 29 (25%)	1000 €—< 2000 €: 15 (13%)
30 – 34: 21 (18%)	Secondary: 38 (33%)		Divorced: 5 (4%)	2000 €—3000 €: 2 (2%)
35 – 39: 11 (10%)	University: 51 (45%)		**Smoking status**	
40 – 45: 7 (6%)	Postgraduate: 2 (2%)		Smoker: 48 (42%)	
			Non-smoker: 66 (58%)	
**Eating habits n (%)**				
**Daily meals**	**Form motives**		**Junk food**	**Form of diet**
1–2: 62 (54%)	Health: 65 (57%)		Not at all: 9 (8%)	Halal: 67 (59%)
3–4: 48 (42%)	Religion: 18 (16%)		Rare: 40 (35%)	No-Halal:47 (41%)
5–6: 4 (4%)	Environmental: 2 (2%)		1–2 times a week: 42 (37%)	**Diet like home**
**Meals taking**	Religion + health: 29( 25%)		3–4 times a week: 18 (16%)	Yes:57 (50%)
Alone: 53 (47%)			5–6 × the week 5 (4%)	No:57 (50%)
In society: 61 (54%)				
**Physical Activity**				
	**Total (** ***N*** ** = 114) Mean ± SD**	**Men (** ***n*** ** = 71) Mean ± SD**	**Women (** ***n*** ** = 43) Mean ± SD**	**Reference Values**
**Total Activity** [Hour/week]	9.86 ± 7.24	10.1 ± 8.07	9.39 ± 55.66	
**Sport Activity** [Hour/week]	0.99 ± 2.17	1.34 ± 2.63	0.41 ± 0.79	
		**n (%)**	**n (%)**	
**Optimal** **Low**		12 (17) 59 (83)	1 (2) 42 (98)	> 2.5 h/week < 2.5 h/week

Half of the participants reported maintaining a diet like their original diet in Syria, and about 59% reported eating halal, yet only 15.8% reported having religious motives regarding their choice of diet. The majority of the participants stated that they regularly consume junk food.

The physical activity data shows that the cohort was only slightly active. The average time spent in physical or leisure-time activity was less than 2.5 h per week for the majority of men (83%) and women (98%).

### Anthropometric and bioelectrical impedance measurements

The average age of men and women was about 28 years (Table 2). Furthermore, the mean BMI for the entire study population was 26.0 ± 4.39 kg/m^2^. More than 63% of men and 34.9% of women had a BMI > 25 kg/m^2^ and can be, thus, classified as overweight or obese. However, men were more likely overweight (42.3%) or obese (21.1%) compared to women (20.9%, 14.0%). On the other hand, the average mean Wc of men (92.8 ± 12.2 cm) and women (79.8 ± 10.9 cm) was within the reference values, although 40% of men and women had a Wc above the WHO reference with > 94 cm for men and > 80 cm for women. 44% of men and 26% of women had a WHR above the reference value (men > 0.90, women > 0.85).

**Table 2:** Anthropometric body composition markers of the study population

	**Total (** ***N*** ** = 114) Mean ± SD**	**Men (** ***n*** ** = 71) Mean ± SD** **n (%)**	**Women (** ***n*** ** = 43) Mean ± SD** **n (%)**	**Reference Values**
**Age [Years]**	**28.2 ± 5.96**	**28.0 ± 5.72**	**28.6 ± 6.38**	
**BMI [kg/m**^**2**^**]**	**26.0 ± 4.39**	**26.9 ± 4.3**	**24.4 ± 4.00**	
Normal		26 (37%)	28 (65%)	18.5–25.0 kg/m^2^ (Normal weight)
Above Ref		30 (42%)	9 (21%)	25.0–30.0 kg/m^2^ (Overweight)
Very high		15 (21%)	6 (14%)	> 30 kg/m^2^ (Obese)
**Wc [cm]**	**87.9 ± 13.3**	**92.7 ± 12.2**	**79.8 ± 10.9**	
Normal		42 (59%)	25 (58%)	M: < 94 / W: < 80 cm
Above Ref		15 (21%)	12 (28%)	M: > 94 / W: > 80 cm
Very high		14 (20%)	6 (14%)	M: > 102 /W: > 88 cm
**WHR**	**0.86 ± 0.08**	**0.89 ± 0.60**	**0.80 ± 0.07**	
Within Ref		37 (56%)	32 (74%)	M: < 0.90 / W: < 0.85
Above Ref		29 (44%)	11 (26%)	M: > 0.90 / W: > 0.85
**ECM/BCM Index**	**0.92 ± 0.16**	**0.82 ± 0.79**	**1.07 ± 0.13**	
Within Ref		69 (97%)	11 (26%)	< 1
Above Ref		2 (3%)	32 (74%)	> 1
**PA [°]**	**6.16 ± 0.85**	**6.65 ± 0.54**	**5.35 ± 0.59**	
Within Ref Below Ref		71 (100%) 0	31 (72%) 12 (28%)	5–9 ^o^ < 5^o^
**BF [Kg]**	**22.2 ± 8.65**	**22.3 ± 8.88**	**22.0 ± 8.33**	
Below Ref Within Ref		0 15 (21%)	0 11 (26%)	dependent on gender, age, and BMI
Above Ref		56 (79%)	32 (74%)	
**LBM [Kg]**	**53.3 ± 10.8**	**59.7 ± 7.71**	**42.6 ± 5.27**	
Below Ref Within Ref Above Ref		0 59 (83%) 12 (17%)	0 43 (100%) 0	dependent on gender, age, and BMI

*BMI* Body mass index, *Wc* Waist circumference, *WHR* Waist-to-hip-ratio, *ECM/BCM* Index Extracellular mass index/body cell mass, *PA* Phase angle, *BF* Body fat, *LBM* Lean Body Mass

Bioelectrical impedance analysis showed that 97.2% of the male participants were in the reference value for the ECM/BCM ratio (< 1). The average PA of men was 6.66° and, thus, within the reference value of 5–9°. None of the male participants were out of the PA reference (< 5%). The situation is different for women. 74.4% of women showed an ECM/BCM index of > 1. The average PA of 5.35° was also significantly lower in women than in men. 27.9% of female participants had a PA of < 5°.

About 21% of men had normal BF, while 79% had a high BF according to reference values. Among the female participants, the proportion with increased BF was relatively similar at 74%. Only 26% of women had a normal BF, while more than 74% had an increased BF content. However, the LBM was within the reference range for most male (83%) and all female subjects.

### Dietary intake

**Fig. 2:**
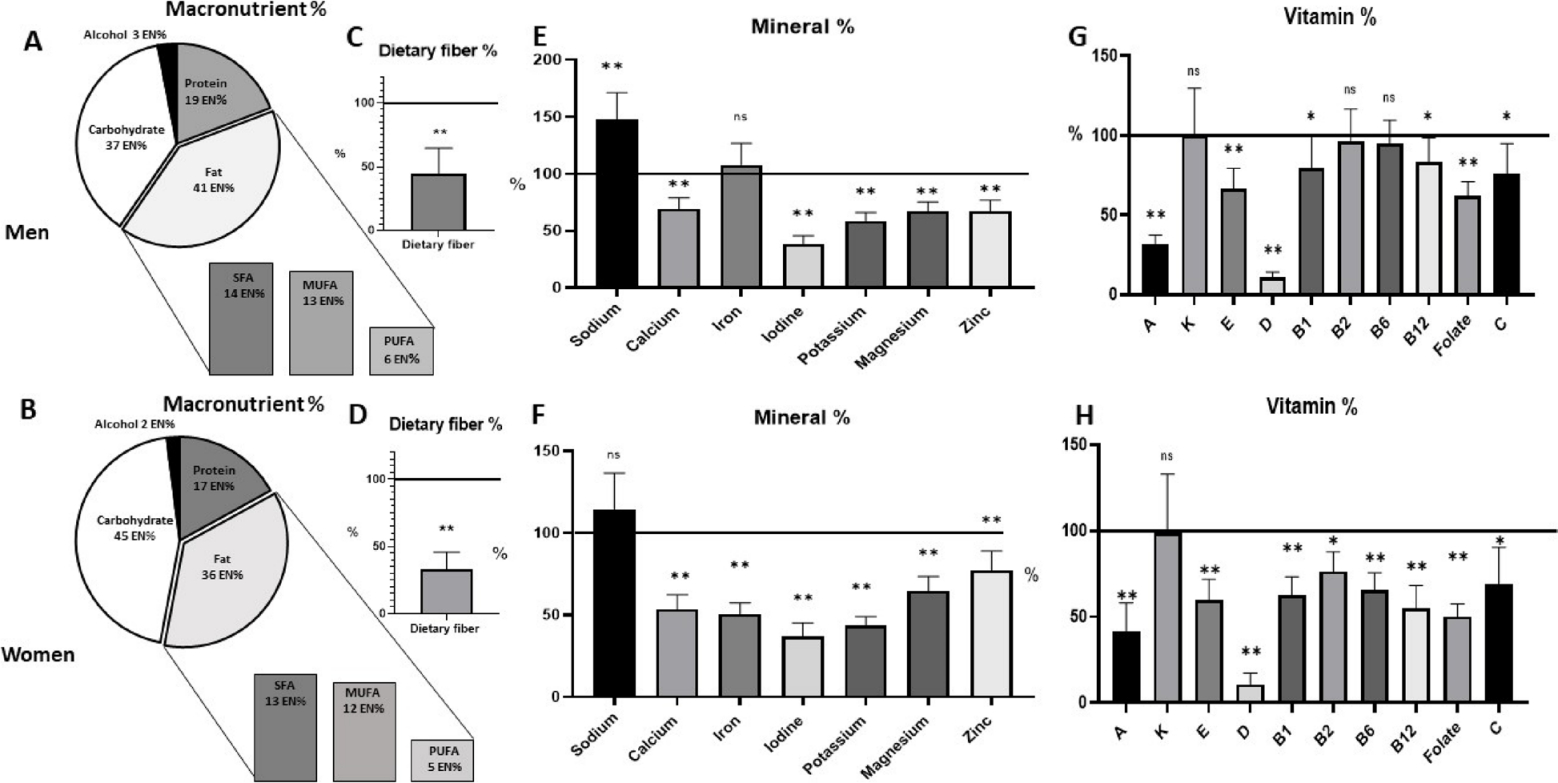
Daily macronutrient (A&B), fiber (C&D), mineral (E&F), and vitamin (G&H) intake of the study population calculated from a three-day dietary record as a percentage from recommendation value by men (*n* = 69) and women (*n* = 41). The mean dietary intake in C-H was compared to the sex-specific D-A-CH references values for nutrient intake. *p* < 0.05, *p* value: One Sample T test, n.s not significant * *p* < 0.005, ** *p* < 0.001

Based on the three-day dietary records, the relative intake of macronutrients related to the energy intake (En%) does not meet the recommendations of the D-A-CH Society (55 En% via carbohydrates, max. 30 En% via fat, and 15 En% via protein). In particular, male participants obtained more energy from fat (40 En%) and protein (19 En%) on average, whereas carbohydrate intake was below recommendations at 38 En% (Fig. 2A). A similar picture—albeit not as pronounced—can be seen among the female participants (38 En% via fat, 17 En% via protein, 45 En% via carbohydrates; Fig. 2B). The qualitative supply of fatty acids also showed that energy intake recommendations for saturated fatty acids (SFA), monounsaturated FAs (MUFA), and polyunsaturated FAs (PUFA) (10 En%, 10–15 En% and 7–10 En%, respectively) were not met. Notably, the male subjects obtained far more SFA than recommended (14 En%), ultimately at the expense of PUFA intake (6 En%). The women also consumed far more SFAs (13 En%) and fewer PUFAs (5 En%) than recommended.

Dietary fiber intake was well below the recommended 30 g/day for both men (20.9 ± 15.9 g/d; Fig. 2C) and women (21.1 ± 23.8 g/d; Fig. 2D). Thus, neither half of the men nor the women reached the recommended intake.

Considering the intake of micronutrients, it becomes clear that the supply of numerous minerals (Fig. 2E/F) and vitamins (Fig. 2G/H) is inadequate and partly critical. This applies to the minerals calcium, iodine, potassium, magnesium, and zinc for both genders and iron in women. In the case of vitamins, the intake of vitamins A, E, and folate in both genders, as well as all B vitamins in women, is occasionally significantly below the intake recommendations. Especially, vitamin D intake was noticeably below the intake recommendation of 20 µg/d in both men (6.67 ± 19.4 µg/d) and women (5.80 ± 18.6 µg/d) in the absence of endogenous synthesis.

### Iron status and hematological parameters

Iron and hematological markers (Table 3) show that men have a significantly better status than women. As expected, the mean Hb value for men (15.5 ± 0.99 g/dl) is significantly higher than for women (13.1 ± 0.89 g/dl). The average iron level in serum is also considerably higher in men (18.3 ± 7.47 µmol/l) than in women (14.8 ± 10.7 µmol/l).

**Table 3:** Iron status and hematological parameters of the study population

Parameters	Total (*N* = 114) Mean ± SD	Men (*n* = 71) Mean ± SD n (%)	Women (*n* = 43) Mean ± SD n (%)	Reference Values
**Hemoglobin [g/dl]**	**14.6 ± 1.51**	**15.5 ± 0.99**	**13.1 ± 0.89**	
Above Ref Within Ref Below Ref		1 (1%) 66 (93%) 4 (6%)	0 38 (88%) 5 (12%)	M: 14–18 g/dl / W: 12–16 g/dl M: < 14 g/dL / W: < 12 g/dL (Anemia)
**Iron [µmol/l]**	**16.9 ± 8.93**	**18.3 ± 7.47**	**14.8 ± 10.7**	
Above Ref Within Ref Below Ref		10 (14%) 59 (83%) 2 (3%)	2 (5%) 37 (86%) 4 (9%)	M: 10.6–28.3 µmol/l / W: 6.60–26.0 µmol/l M: < 10.6 μmol/L / W: < 6.6 μmol/L (Deficiency)
**MCV [fL]**	**84.6 ± 11.2**	**85.2 ± 8.59**	**83.5 ± 14.6**	
Within Ref Below Ref		52 (73%) 19 (27%)	34 (79%) 9 (21%)	83–103 fL < 83 fl (ID anemia)
**Ferritin [µg/l]**	**83.5 ± 72.7**	**113 ± 74.3**	**34.2 ± 31.8**	
Within Ref Below Ref		69 (97%) 2 (3%)	20 (47%) 23 (53%)	M: 30–400 µg/l / W: 15–150 µg/l M: < 30 µg/l / W: < 15 µg/l (Depleted iron stores)
**Transferrin [g/l]**	**2.89 ± 0.43**	**2.80 ± 0.35**	**3.03 ± 0.50**	
Above Ref Within Ref		0 71 (100%)	2 (5%) 41 (95%)	> 4.0 g/l (ID) 2.0–4.0 g/l
**Transferrin saturation [%]**	**23.2 ± 10.8**	**26.2 ± 10.9**	**18.3 ± 8.87**	
Within Ref Below Ref		51 (71%) 11 (16%) 9 (13%)	19 (44%) 7 (16%) 17 (40%)	> 20% 16–20% (Insufficient iron supply) < 16% (ID)

Based on the hemoglobin (Hb) levels, only a small proportion of male (5.6%) and female (11.6%) participants can be classified as anemic. However, other hematological markers for anemia that are not solely dependent on iron intake show evidence of anemia. Given the mean corpuscular cell volume (MCV), both men (28.8%) and women (20.9%) were below the cut-off for iron deficiency (ID) anemia (< 83.0 fl).

Depleted iron stores (serum ferritin < 15 µg/L in women and < 30 µg/L in men) were observed in more than half of the women (53.5%) but only in 2.8% of the men. The transferrin saturation also clearly indicates a marginal iron status in the majority of women, 56%, and almost 28% of men. While 16% of the female subjects showed an insufficient iron supply (transferrin saturation 16–20%), close to 40% observed even a ID (transferrin saturation < 16%).

### 25-OH-Vitamin D status

**Fig. 3:**
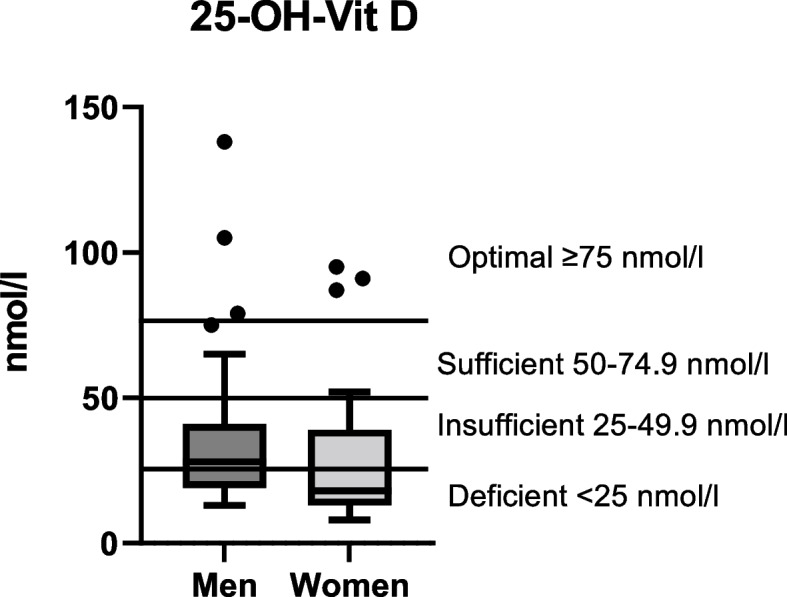
Serum 25-OH-Vitamin D concentrations in men (*n* = 69) and women (*n* = 41)

The 25-OH-Vit D status of the present study cohort was critical, with mean serum 25-OH-Vit D concentrations of 33.9 ± 21.3 nmol/L in males and 26.8 ± 21.6 nmol/L in females (Fig. 3). Sufficient (50–74.9 nmol/L) or optimal (> 75 nmol/L) 25-OH-Vit D levels could only be observed in 12%/6% of males and 2%/7% of females, respectively. The majority of the subjects had an unfavorable 25-OH-Vit D status. Also, we identified considerable gender differences. Female participants showed an even worse 25-OH-Vit D status than men. 38% of the men and 21% of the women showed an insufficient 25-OH-Vit D status (25–49.9 nmol/L), while 44% of the men and 70% of the women were in the deficient 25-OH-Vit D range (< 25 nmol/L).

### Homocysteine

**Fig. 4:**
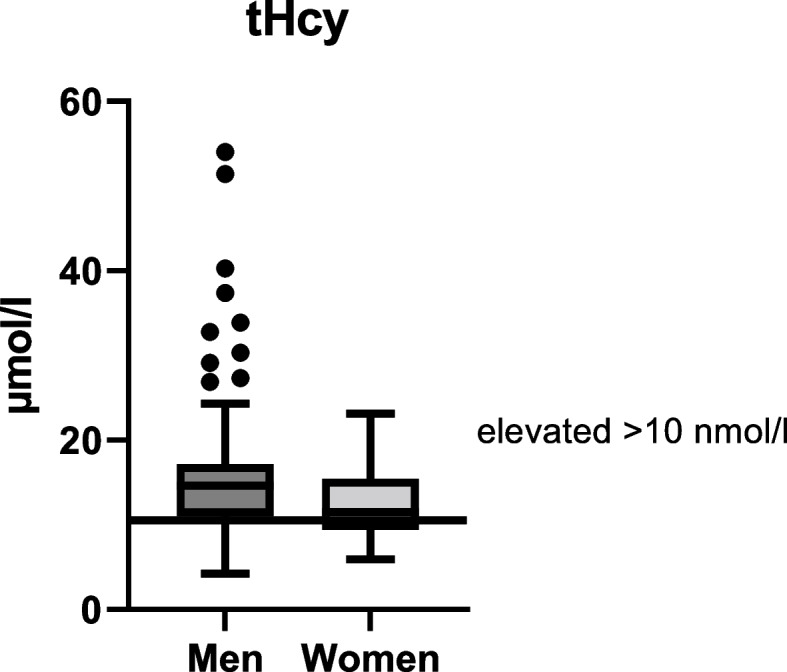
Plasma total homocysteine (tHcy) concentration in men (*n* = 69) and women (*n* = 41)

The mean plasma tHcy concentration of female subjects was 12.6 ± 4.17 µmol/L and 16.8 ± 9.44 µmol/L in male subjects (Fig. 4). 83% of men and 67% of women were above the tHcy cutoff (> 10 µmol/L). Nearly 25% (*n* = 17) of the male subjects showed very high tHcy levels > 18 µmol/L.

### Glucose and lipid metabolism

The subjects' fasting glucose and insulin levels were primarily within the normal range. Especially in male subjects, however, the measured insulin levels showed signs of insulin resistance (IR) in one-fifth of the subjects. Elevated insulin levels were less common in women. In 14% of the male and 12% of the female subjects, the insulin levels were in the range > 15–20 µU/ml, which points to a possible IR. A probable IR (insulin level > 20 µU/ml) was still found in 7% of the men. When looking at the ratio between fasting glucose and insulin using the HOMA-IR index, it becomes even more evident that many subjects of the present cohort exhibit IR. 19.7% of men show a possible IR (HOMA-IR 2–2.5), and as many as 35.2% have likely IR. Among the female participants, 16.3% and 24.6% are within the range of possible and likely IR, respectively.

The serum lipid profile shows that mainly men are hyperlipidemic. While more than 40% of the male subjects had elevated LDL cholesterol levels (> 130 mg/dl), almost a quarter had elevated triglyceride levels (> 150 mg/dl). Cholesterol and triglyceride levels above the reference values for hyperlipidemia were observed in only a few females (Table 4).

**Table 4:** Biomarkers of glucose and lipid metabolism of the study population

Parameters	Total (*N* = 114) Mean ± SD	Men (*n* = 71) Mean ± SD n (%)	Women (*n* = 43) Mean ± SD n (%)	Reference Values
**Glucose [mg/dL]**	**86.3 ± 6.55**	**87.1 ± 6.84**	**85.0 ± 5.89**	
Above Ref Within Ref		2 (3%) 69 (97%)	0 43 (100%)	> 100 mg/dl (prediabetes, DM II) 70–99 mg/dl
**Insulin [μU/mL]**	**10.4 ± 5.08**	**10.1 ± 5.40**	**9.32 ± 4.37**	
Above Ref Above Ref Within Ref		5 (7%) 10 (14%) 56 (79%)	1 (2%) 5 (12%) 37 (86%)	> 20 μU/mL (likely IR) > 15–20 µU/ml (possible IR) ˂15 μU/mL
**HOMA-IR index**	**2.23 ± 0.92**	**2.38 ± 1.20**	**1.99 ± 0.99**	
Within Ref Above Ref Above Ref Above Ref		30 (42%) 14 (20%) 25 (35%) 2 (3%)	25 (58%) 7 (16%) 11 (26%) 0	< 1.0 (IR unliklely) 2.0—< 2.5 (possible IR) > 2.5—5.0 (likely IR) > 5.0 (type-2 diabetes)
**Total cholesterol [mg/dl]**	**184 ± 36.5**	**186 ± 41.3**	**179 ± 26.5**	
Within Ref Above Ref		47 (66%) 24 (34%)	32 (74%) 11 (26%)	> 200 mg/dl < 200 mg/dl
**Triglyceride [mg/dl]**	**108 ± 75.5**	**123 ± 86.6**	**84.3 ± 42.9**	
Within Ref Above Ref		54 (76%) 17 (24%)	40 (93%) 3 (7%)	˂150 mg/dl > 150 mg/dl
**HDL [mg/dl]**	**48.8 ± 11.0**	**45.0 ± 9.66**	**55.1 ± 10.4**	
Below Ref Within Ref		25 (35%) 46 (65%)	13 (30%) 30 (70%)	M: < 40 / W: < 50 mg/dl M: > 40 / W: > 50 mg/dl
**LDL [mg/dl]**	**120 ± 29.2**	**125 ± 31.9**	**113 ± 22.3**	
Within Ref Above Ref		41 (58%) 30 (42%)	34 (79%) 9 (21%)	< 130 mg/dl > 130 mg/dl

### Socio-demographic factors and eating attitudes influencing nutritional/health status

The associations between sociodemographic factors, eating attitudes, and nutritional/health status, and markers of poor prognostic nutritional/health status (i.e., BMI, Wc, BF %, Triglyceride, LDL, HDL, tHcy, and 25-OH-Vit D) were evaluated using multiple linear regression to investigate association with asylum duration. A positive correlation between tHcy ​​and asylum duration was found for refugees who lived in Germany for less than one year (Table 5). This is due to the fact that they had higher tHcy ​​values ​​than refugees who lived in Germany for more than three years (β = 0.169, *p* = 0.030). No other correlations between asylum duration and the rest of the parameters could be found, as shown in Table 5.

**Table 5:** Multiple linear regression analyses of asylum duration and age with nutritional and health status of the study population

**Dependent variables According Asylum duration**	**Model 1**			**Model 2**			**Model3**		
	**R**^**2**^	**β (Cl%)**	***P* value**	**R**^**2**^	**β (Cl%)**	***P value***	**R**^**2**^	**β (Cl%)**	***P* value**
**BMI [kg/m**^**2**^**]**	0.084	-0.229 (-0.17, -0.006)	0.360	0.270	-1.75 (-0.14, 0.007)	0.076	0.361	-0.092 (-0.12, -0.04)	0.392
**Wc [cm]**	0.059	-0.54 (-0.09, 0.06)	0.621	0.409	0.003 (-0.60, 0.62)	0.973	0.453	0.036 (-0.002, 0.11)	0.057
**BF [%]**	0.009	-0.113 (-5.63, 1.85)	0.319	0.286	-0.072 (-4.450, 2.02)	0.459	0.368	0.004 (-3.40, 3.53)	0.970
**Triglyceride [mg/dl]**	0.046	0.048 (-2.11, 0.33)	0.661	0.124	0.059 (-0.191, 0.338)	0.583	0.279	0.116 (-0.13, 0.42)	0.302
**HDL [mg/dl]**	0.043	-0.054 (-7.05, 4.24)	0.622	0.230	-0.061 (-6.73, 3.58)	0.546	0.345	-0.047 (-6.69, 4.23)	0.656
**LDL [mg/dl] **	0.043	-0.213 (-29.5, 0.39)	0.056	0.094	-0.195 (-28.2, -1.14)	0.076	0.183	-0.225 (-31.5, -0.73)	0.061
**tHcy [µmol/l]**	0.041	0.219 (0.001-0.44)	**0.049**	0.104	0.219 (0.004-0.44)	**0.046**	0.169	0.262 (0.03-0.50)	**0.030**
**25-OH-Vit D [nmol/l]**	0.028	0.188 (-0.44, -0.56)	0.093	0.122	0.209 (-0.006, 0.58)	0.055	0.200	0.179 (-0.073, 0.56)	0.130

Model 1: Dummy Asylum duration V1(ref): >3 years, V2: <1 years.

Model 2: Model 1 + Sex + Age.

Model 3: Model 2+ Marital status, Smoking status, Housing, Personal monthly net income, Daily meals, Form motives, Junk food, Form of diet, Diet like home.

## Discussion

Eventhough there have been initial studies on the nutritional situation of Syrian refugees in Germany in recent years [25, 39], this is the first study to assess the individual nutritional situation in a cohort of Syrian refugees in Germany by integrating dietary intake in combination with markers of the nutritional and health status. Together with the previous findings on dietary behaviour [25, 39], this approach contributes to a better understanding of the nutrition and health situation of Syrian refugees, which is a prerequisite to find ways to prevent malnutrition with all its associated health, sociodemographic, and economic consequences.

The group of Syrian refugees examined was small, 114 individuals, but comparable in terms of sociodemographic background (age, asylum duration, educational level, income) to the general situation of Syrian refugees in Germany as reported by the Federal Office for Migration and Asylum (BAMF) [40]. The nutritional situation of the existing cohort can be classified as unfavorable overall. The macronutrient supply was unbalanced, characterized by a high intake of fat and protein, especially in men, and an insufficient intake of carbohydrates. For example, relative energy intake recommendations of the German Society for Nutrition of 15 En% protein, 30 En% fat, and 55 En% carbohydrates were not achieved by either men or women. The quality of the nutrient supply was also unbalanced. Accordingly, the intake of foods with complex carbohydrates and fibers was low. The qualitative fat intake also did not match the recommendations. Recommended intakes of SFA (≤ 11- ≤ 7 En%), MUFA (10–25 En%), and PUFA (6–11 En%) were not met by either men or women [41]. Instead, fatty acid intake was dominated by SFA and poor in PUFAs. However, the pattern of relative macronutrients and absolute fiber intake roughly matched that of the German population [42].

The intake of numerous micronutrients was also below the reference values ​​for intake recommendations [35]. This was particularly evident in women, for whom all minerals and vitamins, except for sodium and vitamin K, were ingested in quantities that were, in some cases, significantly below intake recommendations. The situation for men, in contrast, was only slightly better. The Vitamin D supply was marginal in both sexes.

There is limited data on the nutritional situation of Syrian refugees. The few published studies focused on Syrian refugee cohorts living in the “Middle East,” such as Lebanon [43], Turkey [8], or Iraq [44]. However, since the living conditions and food availability in Middle Eastern countries are fundamentally different from those in Germany or other Western European countries, these data are hardly comparable to our current data.

Compared with an omnivorous mixed diet of the German population, several parallels in the supply of micronutrients exist [45, 46]. For example, the supply of iron (in women), iodine and Vitamin D, and folate is also unfavorable [45, 46]. However, for other nutrients, the supply in the existing cohort of Syrian refugees is inferior than in the German population (especially for calcium, magnesium, zinc, potassium, and vitamins B1, B2, B6, and B12).

The observed unbalanced macronutrient distribution and poor micronutrient supply are the results of a highly refined, energy-dense, hyponutrient food pattern. Many foods that generally contribute to a desirable supply of micronutrients were not consumed or only in small amounts (data not shown). Most test subjects also state that they eat junk food several times per week. It should not be forgotten that the tool used to log the diet only covers three days on which the test subjects may not have eaten their usual diet. It is also possible that some test subjects consciously or unconsciously over- and underreported certain foods (or their amounts), a frequent phenomenon of nutritional self-reports [40, 47]. Further limitations of the three-day nutrition records are discussed in the limitation section.

Thus, in light of the limited data on nutrient intakes from dietary protocols, a joint consideration with the status of some critical micronutrients is much more valid. The insufficient dietary intake of iron in women was also reflected in iron status markers and hematological parameters. Although only a smaller proportion of the female participants showed clear signs of anemia such as reduced hemoglobin (11%) or MCV (21%) levels, more than half of the women showed serum ferritin levels and a transferrin saturation indicative of decreased iron stores and an insufficient iron intake. Poor iron supply and a high incidence of marginal iron stores or ID, with or without anemia, in premenopausal women is a nutritional problem known worldwide, which also occurs in industrialized countries [48–51]. In this context, the iron supply of the existing cohort of Syrian refugees is not surprising. However, the women in the current study had lower serum ferritin levels than young women in comparable cohorts from the Netherlands, Poland, France, or Spain [52, 53]. The iron supply situation of the young female Syrian refugees should be improved by consuming iron-rich foods with highly bioavailable iron (i.e., heme–iron), otherwise, there is a high risk of developing ID anemia with all its physiological consequences, including an increased risk for fetal developmental disabilities in a possible pregnancy [54].

The Vitamin D status in the present cohort can be classified as insufficient in males and deficient in women. Three main reasons are responsible for this poor Vitamin D status: i) low exposure to sunlight ii) limited intake of Vitamin D-rich foods (i.a., mainly fish), and iii) no or little intake of Vitamin D supplements or fortified foods. The insufficient 25-OH-Vit D status of the present cohort is likely the result of an insufficient self-synthesis and, thus, not a nutrition-specific problem. First, the exposure to ultraviolet radiation is limited in countries of the northern latitudes like Germany, especially during the winter months. Second, numerous women from the present cohort are wearing headscarves (veil) and clothes that largely cover the skin due to religious or cultural tradition, which hampers the skin production of Vitamin D [55]. Third, we observed a borderline low Vitamin D intake, which is consistent with a recent study that found a low fish consumption, as the only relevant Vitamin D source, in Syrian refugees [25]. Despite the global Vitamin D deficiency pandemic [56], it is known that Vitamin D deficiency is even more common in non-western refugees in Western countries compared to the host population [57, 58]. Non-western refugee cohorts often show up to 50% Vitamin D deficiency. In the existing cohort, the proportion of women with severe Vitamin D deficiency was 70%. Together with the low dietary calcium intake, the present cohort is at risk for developing osteopenia and osteoporosis, besides the risk of Vitamin D deficiency for common cancers, autoimmune diseases, hypertension, and infectious diseases [56].

In addition to the suboptimal nutritional situation or as a consequence, many Syrian refugees also have abnormalities in their health that increase the risk of metabolic disturbances and nutrition-related diseases such as cardiovascular diseases (CVD) or T2D. This circumstance is striking for a young (and otherwise healthy) cohort. For example, elevated tHcy levels (> 10 nmol/l) were found in the majority of the subjects, whereas hyperhomocysteinemia is particularly evident in the male subjects. Around 38% of men in this study had mild hyperhomocysteinemia (Hcys > 15 μmol/l) compared to 7% in adult Germans in the same age group [59]. Many epidemiologic and case-controlled studies have demonstrated an association with hyperhomocysteinemia and CVD as well as stroke [60]. The B vitamins folate, B12, and B6 play a key role in homocysteine ​​metabolism. The insufficient supply of folate, B12, and B6 is also associated with hyperhomocysteinemia [61]. In women, in particular, the supply of B12, B6, and folate is inadequate, whereas men showed a poor intake of vitamin B12 and folate. Another explanation might be the psychological stress of the refugees, which is significantly and positively correlated to the tHcy ​​concentration [62, 63].

Furthermore, the serum lipid levels of the subjects show a frequent occurrence of dyslipidemia. About one-third of men have elevated total and LDL cholesterol levels, which are accompanied by reduced HDL levels. Triglyceride levels are also elevated in 24% of male subjects. In female subjects, hypercholesterolemia is not as severe, but around a quarter of the female participants still have abnormal serum lipid levels. The glucose and insulin levels of the subjects are not abnormal on average, but when the HOMA-IR index is used, it is evident that insulin resistance (prediabetes) is likely in > 50% of men and > 40% of women. Thus, many subjects of the present cohort can be classified as prediabetic with an increased risk to manifest T2D. A meta-analysis showed a high prevalence of T2D among Syrian refugees in Syria's neighboring host countries [64]. Syria is considered to be one of the countries with the highest prevalence of T2D worldwide. It is estimated that by 2022, nearly one-fifth of the Syrian population aged 25 years or older have T2D [65].

Considering the anthropometric markers BMI and Wc, more than a third of women and more than half of men in the present cohort are overweight or even obese. Around three-quarters of all subjects even have increased BF values. The mean BMI of the group was 26.0 ± 4.39 kg/m^2^, which is comparable to that in Germany (26.85 kg/m^2^) [52]. Reasons for the high prevalence of overweight/obesity in migrant populations are nutrition-associated (high-fat, SFA-rich; energy-dense foods), inactive lifestyle (low physical activity), stress exposure as well as sociodemographic factors (i.e., low income) [66]. Around a quarter of women have a PA of < 5°, which indicates poorly nourished cells and cell integrity and is therefore also consistent with the observed poor nutrient supply situation. However, the prognostic value of PA for malnutrition is discussed controversially [67].

Based on the regression analysis of health parameters and residence duration, we conclude that duration of residence does not have an impact on prognostic health parameters except tHcy. As stay of residence was positively associated with lower tHcy values, it might be tempting to speculate that a longer residence may be associated with a reduced B vitamin intake involved in tHcy metabolism. Likewise, psychological stress after migration as well as an increasingly sedative lifestyle may also negatively affect tHcy levels. In contrast to previous studies [13, 15, 18], we did not observe any association between the duration of stay in Germany and BMI, body fat, or blood lipids. This inconsistency may be explained by the relatively short duration of stay in this study as half of the participants had lived in Germany for less than three years. Given the lack of data on refugee dietary patterns and their impact on various indicators of nutritional status, the study recommends that future studies should focus on deepening understanding of the associations between refugee dietary patterns and behaviors, especially those with religious backgrounds, that may impact nutritional status assessments over time.

### Limitations

The study has some potential limitations. First, the study cohort is not formally representative of the population of Syrian refugees in Germany, since the sample was recruited in only one city in Germany. Second, the study cohort has a small sample size. Therfore, our results cannot be simply be extrapolated to all Syrian refugees in Germany. The response rate during the recruitment of study subjects was low as many of the potential participants had political and social concerns despite the anonymity of their participation. The assessment of nutrient intake via three-day dietary records may be somewhat biased due to potential over- or underreporting by participants and the fact that the diet during the three days may not be representative of the subject’s usual diet. In addition, the translation of the questionniares into Arabic may be another source of error. In particular, the over- and underreporting are evident considering a) the discrepancy between nutrient deficiency and overweight and b) the difficult motivation of the refugees to participate in the study. In addition, the estimation of nutrient amounts in foods and food products using software-based calculation tools is vulnerable to potential errors in nutrient composition found in the food databases of the nutrient intake calculation software. Although BIA is an accepted tool for estimation of body composition [68–70], this method also has its limitations, as it is susceptible to confounding factors such as vigorous physical activity, food intake, or hydration status [68, 71, 72]. However, all measurements were conducted in the morning after overnight fasting according to standardized procedures.

## Conclusion

The nutritional and health status of the cohort of Syrian refugees in Germany examined in this pilot study is unsatisfactory concerning several nutrients. The unbalanced malnutrition was characterized by a high total fat and SFA intake and an undersupply of critical minerals and vitamins due to highly refined, energy-dense, and hypomicronutrient-rich food choices. However, the various metabolic health markers alone should not to be considered critical since multiple risk factors such as hypercholesterolemia, hypertriglyceridemia, IR, hyperhomocysteinemia, smoking, overweight/obesity, and physical inactivity were present simultaneously. Consequently, many of the Syrian refugees investigated had an increased risk of developing metabolic diseases such as CVD and T2D. The nutritional and health situation of the Syrian refugees examined is similar in many respects to the host population in Germany. Against the background of the high number of refugees from different countries in Germany, which is currently increasing significantly due to the war in Ukraine, the issue of healthy nutrition as a prerequisite for health and the absence of nutrition-related diseases is of great importance and should be investigated in more detail in future studies, also to avoid high costs for our health care systems. The findings from such studies should be incorporated into integration policies to increase health awareness among immigrants.

## Data Availability

The datasets used and analyzed during the current study are available from the corresponding author on reasonable request.

## Abbreviations

BMI: Body mass index
Wc: Waist circumference
WHR: Waist to hip ratio
ECM/BCM Index: Extracellular mass index/body cell mass
Pa: Phase angle
BF: Body fat
ID: Iron deficiency
CVD: Cardiovascular disease
T2D: Type 2 Diabetes mellitus
IR: Insulin resistance
SFA: Saturated fatty acid
MUFA: Monounsaturated fatty acid
PUFA: Polyunsaturated fatty acid
En%: Energy-%, tHcy: total homocysteine
